# I see what you are saying

**DOI:** 10.7554/eLife.17693

**Published:** 2016-06-09

**Authors:** Gregory B Cogan

**Affiliations:** Department of Biomedical Engineering, Duke University, Durham, United Statesgregory.cogan@duke.edu

**Keywords:** lip movements, speech, language, oscillations, magnetoencephalography, electroencephalography, Human

## Abstract

The motor cortex in the brain tracks lip movements to help with speech perception.

**Related research article** Park H, Kayser C, Thut G, Gross J. 2016. Lip movements entrain the observers’ low-frequency brain oscillations to facilitate speech intelligibility. *eLife* **5**:e14521. doi: 10.7554/eLife.14521

**Image** Speech is easier to understand if you can watch the speaker’s lips

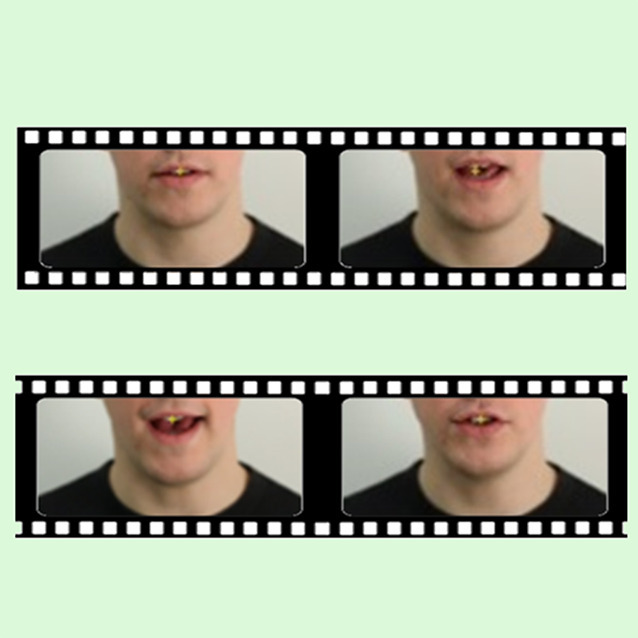

In the mid-1940s, the psychologist Alvin Liberman went to work with Franklin Cooper at the Haskins Laboratories in New Haven, Connecticut. He initially set out to create a device to turn printed letters into sounds so that blind people could ‘hear’ written texts (Liberman, 1996). His first foray involved shining a light through a slit onto the page in order to convert the lines of each letter into light and then into frequencies of sound. Liberman and colleagues reasoned that with enough training, blind users would be able to learn these arbitrary letter-sound pairs and so be able to understand the text.

The device was a spectacular failure: the users performed slowly and inaccurately. This led Liberman and colleagues to the realization that speech is not an arbitrary sequence of sounds, but a specific human code. They argued that the key to this code was the link between the speech sounds a person hears and the motor actions they make in order to speak. This important work led to decades of further research and helped lay the foundation for the psychological and neuroscientific study of speech.

When we watch and listen to someone speak, our brain combines the visual information of the movement of the speaker’s mouth with the speech sounds that are produced by this movement (McGurk and MacDonald, 1976). One of the core problems that researchers in this field are investigating is how these different sets of information are integrated to allow us to understand speech. Now, in eLife, Hyojin Park, Christoph Kayser, Gregor Thut and Joachim Gross of the University of Glasgow report that they have studied this integration by using a technique called magnetoencephalography to record the magnetic fields that are generated by the electrical currents of the brain (Park et al., 2016).

Park et al. presented volunteers with audio-visual clips of naturalistic speech and then asked them to complete a short questionnaire about the speech they heard and saw. In some cases, these clips were manipulated so that the audio did not match the video. In other cases, Park et al. presented a different speech signal to each ear and asked the volunteers to pay attention to just one signal. By analyzing these combinations, they could separate the brain activity that is associated with watching someone speak from the activity that processes the speech sounds themselves.

Park et al. found that a part of the continuous speech stream called the envelope, which is the slow rising and falling in the amplitude of the speech, was tracked in auditory areas of the brain (Figure 1). Conversely, the visual cortex tracked mouth movements. These results are a good replication and extension of previous data recorded from both the auditory domain (Cogan and Poeppel, 2011; Gross et al., 2013; Luo and Poeppel, 2007) and the visual domain (Luo et al., 2010; Zion Golumbic et al., 2013). However, Park et al. extended these findings by asking: what role does tracking the lip movements of a speaker play in speech perception?

**Figure 1.**
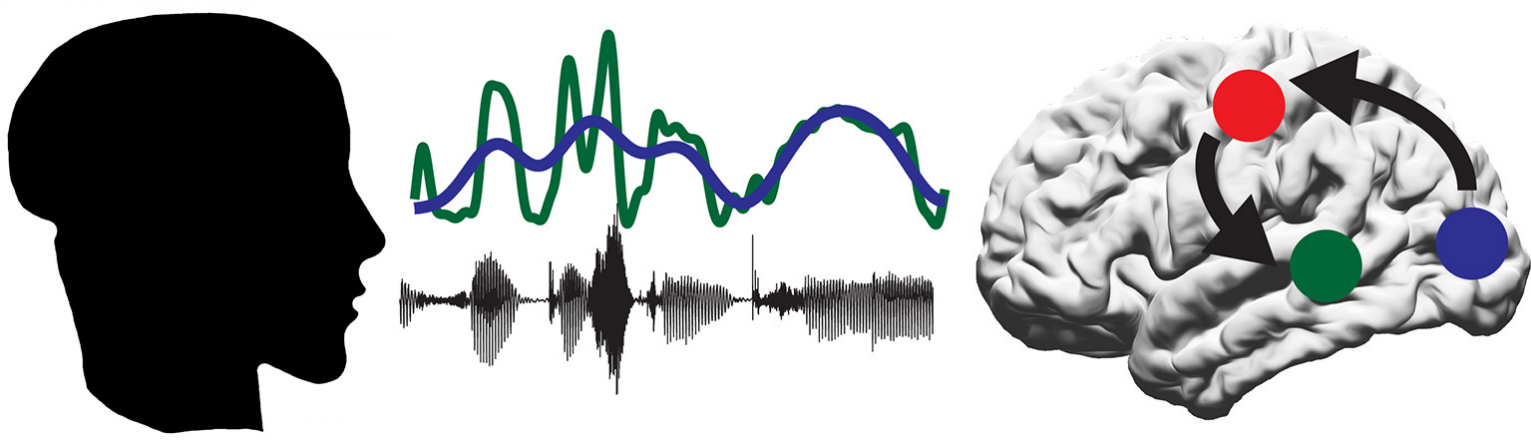
A proposed model for the role of the motor system in speech perception. A person produces speech by the coordinated movement of their articulatory system. The listener hears the sound (black line) and sees the mouth of the speaker open and close (represented by blue line). Some of the information in the sound is contained within the speech envelope (green line). The auditory regions of the brain (green circle) track the speech envelope, while the visual system (blue circle) tracks the visual movements of the mouth. The motor system (red circle) then decodes the intended mouth movement and integrates this with the response of the auditory regions to the incoming sounds.

To learn more about which parts of the brain track the lip movements of the speaker, Park et al. performed a partial regression on the lip movement, envelope and brain activity data to remove the response to sound and focus on just the effect of tracking the lip movements. This revealed two areas of the brain that actively track lip movements during speech. The first area, as found by previous researchers, was the visual cortex. This presumably tracks the lips as a visual signal. The second area was the left motor cortex.

To further establish the role of the motor cortex during speech perception, Park et al. examined the comprehension scores from the questionnaire. These scores could be predicted from the extent to which neural activity in the motor cortex synchronized with the lip movements observed by the participant: higher scores correlated with a higher degree of synchronization. This suggests that the ability of the motor cortex to track lip movements is important for understanding audiovisual speech, suggesting a new role for the motor system in speech perception. Park et al. interpret this finding to suggest that the motor system helps to predict the upcoming sound signal by simulating the speaker’s intended mouth movement (Arnal and Giraud, 2012; Figure 1).

While this is an important first step, it is still not clear how the lip movement tracked by the motor cortex is integrated with the response of auditory regions of the brain to speech sounds. Are mouth movements tracked specifically for ambiguous or difficult stimuli (Du et al., 2014) or is this tracking necessary for perceiving speech generally? Future work will hopefully clarify the specifics of this mechanism.

It is interesting and somewhat ironic that the motor cortex tracks the visual signals of mouth movement, given the early (and unsuccessful) efforts of Liberman and colleagues to help the blind ‘hear’ written texts. Indeed, just as these early researchers proposed, it seems that the link between the motor and auditory system is a key to understanding how speech is represented in the brain.
